# The spatial structure of chronic morbidity: evidence from UK census returns

**DOI:** 10.1186/s12942-016-0057-5

**Published:** 2016-08-24

**Authors:** Peter F. Dutey-Magni, Graham Moon

**Affiliations:** 1 Geography and Environment, University of Southampton, University Road, Southampton, SO17 1BJ UK; 2 Department of Social Statistics and Demography, University of Southampton, University Road, Southampton, SO17 1BJ UK

**Keywords:** Spatial autocorrelation, Spatial dependency, Spatial interaction, Spatial weights, Neighbourhood matrices, Disease mapping, Chronic morbidity, Limiting longstanding illness

## Abstract

**Background:**

Disease prevalence models have been widely used to estimate health, lifestyle and disability characteristics for small geographical units when other data are not available. Yet, knowledge is often lacking about how to make informed decisions around the specification of such models, especially regarding spatial assumptions placed on their covariance structure. This paper is concerned with understanding processes of spatial dependency in unexplained variation in chronic morbidity.

**Methods:**

2011 UK census data on limiting long-term illness (LLTI) is used to look at the spatial structure in chronic morbidity across England and Wales. The variance and spatial clustering of the odds of LLTI across local authority districts (LADs) and middle layer super output areas are measured across 40 demographic cross-classifications. A series of adjacency matrices based on distance, contiguity and migration flows are tested to examine the spatial structure in LLTI. Odds are then modelled using a logistic mixed model to examine the association with district-level covariates and their predictive power.

**Results:**

The odds of chronic illness are more dispersed than local age characteristics, mortality, hospitalisation rates and chance alone would suggest. Of all adjacency matrices, the three-nearest neighbour method is identified as the best fitting. Migration flows can also be used to construct spatial weights matrices which uncover non-negligible autocorrelation. Once the most important characteristics observable at the LAD-level are taken into account, substantial spatial autocorrelation remains which can be modelled explicitly to improve disease prevalence predictions.

**Conclusions:**

Systematic investigation of spatial structures and dependency is important to develop model-based estimation tools in chronic disease mapping. Spatial structures reflecting migration interactions are easy to develop and capture autocorrelation in LLTI. Patterns of spatial dependency in the geographical distribution of LLTI are not comparable across ethnic groups. Ethnic stratification of local health information is needed and there is potential to further address complexity in prevalence models by improving access to disaggregated data.

**Electronic supplementary material:**

The online version of this article (doi:10.1186/s12942-016-0057-5) contains supplementary material, which is available to authorized users.

## Background

The spatial distribution of chronic morbidity at a subnational level attracts considerable policy interest with relevance for health inequalities, health care planning, and resource allocation. Yet, information on the spatial distribution of morbidity is typically scarce with researchers often reverting to data on mortality or using data on health service use. Intelligence on the small area population prevalence of morbidity has tended to focus on cancer incidence and mortality [1, 2], cancer risk factors and screening uptake [3], the prevalence of long-term conditions [4, 5], healthy lifestyles and behaviours [6, 7]. There has also been interest in measuring geographical variations in health needs [8, 9] and underdiagnosis of long-term conditions [10].

The challenges involved in developing small area measures of morbidity have led to a range of techniques known as small area estimation. Model-based approaches to small area estimation rely on the premise that a chosen statistical model accurately predicts the odds of illness for the entire population. They raise a series of challenges in terms of validity. In the absence of systematic procedures guaranteeing optimal model specification and selection, there is a risk that this modelling process will be ill-informed, introducing bias in the resulting estimates. Reviews have argued that assumptions around the treatment of spatial effects introduces a particular element of subjectivity [11, 12, p. 87].

The objective of this paper is to assess spatial dependence between small geographical areas for chronic morbidity. We analyse the geographical distribution of limiting long-term illness (LLTI) across England and Wales, focusing on the spatial structure in morbidity both with and without controls for confounders (mortality and hospitalisation rates). We consider global and local autocorrelation statistics for three types of dependence structures: contiguity, nearest *k*-neighbours and a novel approach building a spatial interaction matrix using origin-destination migration flows. Our analyses are stratified by ethnicity to isolate differences in the spatial structure of morbidity across different population subgroups. This results from existing interest in monitoring health inequities across ethnic groups. It is currently unclear from the literature how homogenous the spatial structure of morbidity is across ethnic groups, especially given the complex interaction with existing processes of residential segregation.

The following background section gives a review of existing knowledge on spatial aspects of health determinants, to inform model selection. Aims and methods are then outlined, with a particular emphasis on concepts used to describe spatial structures. A results section then presents both descriptive statistics and model-based analyses of the geographical distribution of LLTI, introducing mortality, hospital admissions and adjacency matrices as predictors of this structure. The paper then concludes by identifying implications for the routine prediction of morbidity prevalence for different geographical units.

### Existing knowledge on the spatial structure of chronic morbidity

Much of what is known on the distribution of chronic diseases comes from data on validated self-reported health statuses. LLTI has emerged as a very strong predictor both of chronic morbidity and mortality [13–15]. It has also proved instrumental in measuring health inequalities both across socioeconomic categories and space [16–18]. LLTI has been recorded since 1991 in UK decennial censuses in the form of a question asking whether respondent’s day-to-day activities were reduced by a health problem or disability. This information has supported important research into the determinants of health care needs of different populations in different places [19, 20].

The literature provides some information regarding ecological determinants of chronic morbidity and their spatial structure. Analyses have showed that, even once population age and essential demographic confounders are controlled for, adjusted morbidity levels correlate significantly with local socioeconomic characteristics [17], and the remaining between-area heterogeneity is spatially structured [21]. To examine these ‘place effects’, Bentham et al. [22], Martin et al. [23], Senior et al. [16], Shouls et al. [21, 24], Congdon [4, 25, 26] and Stafford et al. [27] have all investigated the association of LLTI prevalence with both individual-level characteristics and area-level contextual variables. Their work has showed that local mortality, unemployment, household overcrowding, ethnic diversity, social renting, proportions of workers employed in mining and other heavy industries all correlated strongly with standardised ratios of LLTI. These confounders often prove to be similar in places that are near to each other (for instance across urban areas), pointing to distinctive underpinning spatial structures.

A variety of processes have been hypothesised to explain this apparent clustering of long-term conditions across places. On the one hand, it is the case with many health outcomes that a residual spatial pattern can subsist even once observable risk factors or confounders are taken into account [28]. On the other hand, research has argued that population migration not only determines the dispersion of communicable disease, but also provides one of the factors driving the spatial clustering of chronic morbidity. The literature has in particular examined ‘health selective’ residential migrations as life course processes of selection [29]. Boyle et al. [30] have produced evidence that Scottish migrants tend to be healthier than non-migrants, and that healthy migrants are likely to travel longer distances. Further evidence supporting the theory of a ‘sorting’ effect of migrations on health has been presented by Norman et al. [31], emphasising the existence of a strong flow of healthy migrants aged 20–59 years towards areas with lower levels of material deprivation. A review by Smith & Easterlow [32] argues that the influence of residential mobility processes on geographical inequalities in morbidity and mortality remains little understood, with mixed results depending on the geographical level of analysis and the health outcomes under consideration. Despite the absence of clear evidence claims for the health sorting effects of migration point to a need to consider how we might use migration data to capture some of the spatial structure in morbidity in a way that proximity may not.

All the above evidence has implications for disease prevalence models. In its most elementary form, model-based small area estimation fits a model predicting the probability of having a given illness as a function of age, sex, and other individual characteristics. This model is then applied to local population estimates and auxiliary data known for every individual residing in a catchment area in order to produce a local prevalence estimate. This amounts to interpolating prevalence levels known at the national level to local populations using a combination of:

- fixed individual-level risk confounders
- spatially varying area-level confounders
- residual unobserved risk (between-area residual heterogeneity in prevalence)

This last component (c) is essential and explains the popularity of multilevel health models in recent decades [33], being one of the preconditions to the model’s unbiasedness. Residuals capture local departures from the overall average which signals, for instance, excess morbidity. This random component avoids assuming for instance that all persons aged 16–24 years have the same prevalence across all areas. This component is difficult to estimate because sample data will typically be small, often well under a few dozen cases. More importantly, the underpinning method assumes that these residuals are independent from one another and often ignores the fact that spatial dependence may persist. Recognising underlying spatial structure makes it possible to borrow information from other areas in order to estimate these components in a more efficient manner (see for instance simulation results by Praseti & Salvati [34]).

More research is needed to understand spatial dependence. Spatial structures have previously been described as the result of ‘the operation of processes in which spatial relationships enter explicitly into the way the process behaves’ [35, p. 24]. They are often understood as functions of distance or spatial adjacency (neighbours). The science of spatial autocorrelation has largely been dominated by Tobler’s First Law of Geography, summarised as ‘everything is related to everything else, but near things are more related than distant things’ [36]. Contiguity methods, such as Queen, Rook or Bishop, and the *k*-nearest neighbours method have traditionally been privileged. Although this standard approach is appealing, there are many more ways in which spatial interaction could be defined. In particular, origin/destination migration flow statistics constitute additional evidence of processes of spatial interaction and therefore between-area dependence. Although using such flow metrics to produce spatial weights has been envisaged before [37, p. 271], they have, to the best of our knowledge, not been applied to empirical investigation to date.

Internationally, most research has tended to demonstrate that there is global spatial autocorrelation in many health outcomes even after age standardisation [38]. This autocorrelation is a sign of spatial similarity in unobserved risk factors [28]. Yet, it remains unclear whether these spatial patterns are homogeneous once we disaggregate by demographic subgroup, and add explicit spatially varying area-level confounders.

This justifies looking further into spatial structures themselves, to inform non-communicable disease mapping methods with a particular focus on the type of constraints placed on the treatment of residual between-place heterogeneity. On the basis of this background we propose to examine the spatial structures of LLTI in a more systematic way, investigating (a) what structures can be uncovered in terms of dispersion, autocorrelation, and contextual effects, (b) whether they are the same across different subgroups (age and ethnicity) and (c) whether they subsist once good area-level covariates are introduced. We aim to address a current gap in knowledge regarding the spatial structure of morbidity in England and Wales, but also to reconsider the specification of disease prevalence models.

## Methods

### Data source

We use 2011 census data on LLTI for England and Wales [39]. Although the quality issues concerning self-assessed health information are well documented [40], a key advantage of using census data lies in the absence of sample size restriction. The 2011 census met a high quality 93 % person coverage rate for England and Wales [41], and thus constitutes a unique source of information to establish prior knowledge on the spatial structure of illness. Census data provide sufficient statistical power to examine model-fitting hypotheses which usually cannot be tested with survey data due to lack of power. This is especially true for small population subgroups such as older people and ethnic minorities, whose representation in health surveys is too weak in comparison to the amount of interest they attract. This reduces risks of model overfitting when using a large number of parameters. With the census coverage survey’s adjustments for nonresponse [42], the final sample size used for this analysis is *n* = 56,075,912.

We examine private households’ returns for question no. 23:

‘Are your day-to-day activities limited because of a health problem or disability which has lasted, or is expected to last, at least 12 months? Include problems related to old age’.

Respondents were able to answer ‘Yes, limited a lot’, ‘Yes, limited a little’, or ‘No’. Throughout this paper ‘LLTI’ refers to strong activity limitations (‘limited a lot’) which has been found to have a better rate of agreement in the post-enumeration Census Quality Survey [40].

The choice of indicator is justified by two main reasons. First, LLTI has become a central indicator to measure inequalities in health and health needs, to the point of being included in most UK household surveys. It underpins indicators such as the Slope Index of Inequality in health, the disability-free life expectancy, as well as gender and ethnicity gaps in health. These have been for a number of years to inform health service policy aiming to reduce health inequalities [43]. Several of the Office for National Statistics’ products estimate these indicators for local authorities [44, 45], and efforts have been made to publish them for smaller units [20]. Second, although self-reported, the LLTI health status correlates with important indicators of chronic conditions. In addition to being a good predictor of health service use [19], it is also a strong predictor of diagnoses as defined in the International Classification of Diseases [46], although evidence tends to suggest that LLTI tends to underestimate morbidity compared to clinical records or the more demanding SF-36 tool [14].

### Statistical methods

This paper aims to address gaps in knowledge regarding the spatial structure of chronic morbidity and provide evidence relevant to build small area estimation models. We explore spatial heterogeneity in the odds of LLTI at a scale for predictions to be feasible for small ethnic groups: local authority districts (LADs), areas with populations ranging from 34,000 to 1.1 million inhabitants; and middle layer super output areas (MSOAs), census geographical units averaging 7700 residents. Standard descriptive statistics are used to characterise the spatial structure in odds: variance and autocorrelation. A series of models then analyse this structure conditionally on contextual data (mortality, hospitalisations), using a typical logistic binomial parameterisation:

1 $$\begin{aligned} \mathrm {log}\left( \dfrac{y_{id}+.5}{n_{id}-y_{id}+.5}\right) = \mu _{id}+\upsilon _d = \varvec{x}_{id} \varvec{\beta } + \upsilon _d \end{aligned}$$

where $y_{id}$ is the number of individuals belonging to a cross-classification *i* of gender (1, 2), age group (‘0–15’, ‘16–49’, ‘50–64’, ‘65+’), and ethnic group (‘White’, ‘Mixed’, ‘Asian’, ‘Black’, ‘Other’) reporting an LLTI in a given area *d*. $n_{id}$ denotes the total number of residents of private households at risk for this same cross-classification, $\mu _{id}$ the conditional mean log-odds of having an LLTI (fixed part of the model), $\varvec{\beta }$ a column vector of fixed effect coefficients, and $\varvec{x}_{id}$ a vector of covariates known for all individuals: age, sex and ethnicity dummy variables, as well as area-level characteristics tested in this paper. Random intercepts $\upsilon _d$ are realisations of a random variable $\varvec{\upsilon }$ of mean zero and variance $\sigma ^2$. We add 0.5 to both the numerator and the denominator of odds to produce ‘empirical logits’, addressing bias arising from the presence of null denominators [47, 48].

Models are estimated using Laplace approximation with the R package lme4 [49, 50]. We use classical model selection techniques; likelihood ratio tests, the Akaike Information Criterion (AIC) and regression coefficient significance. During model selection, attention was also paid to $\sigma ^2$, the variance of random effects $\varvec{\upsilon }$, which reflects the between-area dispersion in prevalence that is not attributed to differences in covariates included in the fixed part. The reason why $\sigma ^2$ is used as a decision factor is that it plays a considerable part in the efficiency of estimation [51]. Approximations of the mean squared error of prediction developed by Prasad and Rao [52] and extended to log-linear models [53, 54] show that the main determinant of prediction error is the size of $\sigma ^2$ compared to the within-group variance. By attempting to reduce $\sigma ^2$ as much as possible, we focus on improving the predictive power of the fixed part. This is important when conducting small area estimation in real world conditions because residuals $\upsilon _d$ will often be estimated with very small sample sizes and therefore subject to substantial error. A strong fixed part $\mu _{id}$ is likely to produced better predictions overall.

### Defining ‘spatial structures’

Global spatial autocorrelation is measured using the Moran’s *I* statistic, with a random permutation test for significance testing [55]. Local autocorrelation of regression residuals is also examined using a local indicator of spatial autocorrelation (LISA) [56] and the Moran scatterplot [57]. These are used to detect significant leverage of one set of neighbours on the global (average) level of autocorrelation, thereby signalling a cluster of high or low similarity.

Four types of adjacency matrices were tested (see Table 1). **L.A** and **M.A** follow the standard approach and were generated using the spdep package [58, 59] and boundary shapefiles [60]. They are based on the Queen method: areas were coded as neighbours in the adjacency matrix if their digital boundaries shared at least one point or if two of their respective points were separated by less than 500 m. This ensures for instance that London boroughs separated by the River Thames are coded as neighbours. The final matrix was edited manually to attach islands to mainland neighbours and verify that no area was left without neighbours. **L.B***k* and **M.B***k* were produced using the *k*-nearest neighbours method for *k* values of 2–10, with the view of determining an optimal *k*. All matrices were row-standardised, a procedure that is traditionally used to ensure the positive-definitiveness of correlation matrices in various conditional autoregressive models when spatial weight matrices are not symmetric [61].

**Table 1:** Standardised proximity matrices tested in this paper for between-LADs and between-MSOAs autocorrelation

Matrix identifiers		Method of construction
LADs	MSOAs	
**L.A**	**M.A**	Contiguity matrix (with isles attached to the mainland) [spdep + manual adjustments]
**L.B** *k*	**M.B** *k*	*k*-nearest neighbours (based on Euclidian distances between population centroids) [spdep]
**L.C** *k*	–	Up to *k* LADs from which most new residents originate [62], binary weights
**L.D** *k*	–	Up to *k* LADs from which most new residents originate [62], proximity weights *k*, …, 3, 2, 1

For LADs, additional matrices **L.C** and **L.D** were built using migration flows as a proxy for spatial dependence. There are good reasons why areas further apart could be more closely related to each other given the UK’s urban and rural structure. Proximity is not the only reason why risk factors would be more alike in areas. Intra-national origin-destination migration data published by the Office for National Statistics [62] were used to construct spatial weights based on the intensity of flows (see $\texttt{R}$ syntax in Additional file 1). For every LAD, we defined neighbours as the *k* areas from which the most migrants originate, based on the ratio of the total migrants they contributed relative to their respective population sizes. In other words, neighbours are not just those that send most migrants to a given district, they are the ones for which these migrants represent the highest proportion of their respective populations. This is to ensure a fair weighting across all LADs in the process of averaging odds of LLTI, and especially ensure that the resulting neighbours would not systematically be the biggest LADs. If a district *A* sent a large number of migrants to district *B*, but this flow in fact represented a very modest volume relative the entire population of *A*, it would seem excessive to use the odds of poor health of the entirety of district *A* as a smoothing reference for district *B*.

Sensitivity analyses on a subset of LADs suggested that selecting neighbours who send the highest number of migrants or those who send migrants flows which represent the highest proportion of their total population did not alter the eventual list of neighbours substantially. Further analyses (see Additional file 2) were conducted to establish whether origins and destinations differed substantially depending on the age of migrants. Results showed that excluding younger migrants did not have a strong influence on the resulting matrices. However, we hypothesised that student migrations, which are only temporary, are likely be less determinant of the structure of LLTI than other types of migrations taking place across life. Final spatial weight matrices were therefore generated exclusively based on flows for migrants aged 30 years and over.

## Results

### Descriptive characteristics

Overall across English and Welsh LADs, the mean odds of LLTI is $9.23 \times 10^{-2}$ (equivalent to an 8.40 % mean prevalence) with a variance of $7.01 \times 10^{-4}$, equivalent to a 28.7 % coefficient of variation. This masks huge differences across subgroups. Examining age, Table 2 suggests that the between-area variance in odds of LLTI among older groups is several hundred times that of younger groups. This implies that the level-2 variance is expected to be higher for older age groups. Much of this effect can be attributed to the higher prevalence of LLTIs among older populations; larger odds by definition have larger variances. Coefficients of variation reported in Table 3 confirm this; relative to the average of all odds across England and Wales, the dispersion is of the same order of magnitude across age and gender groups for White populations.

**Table 2:**
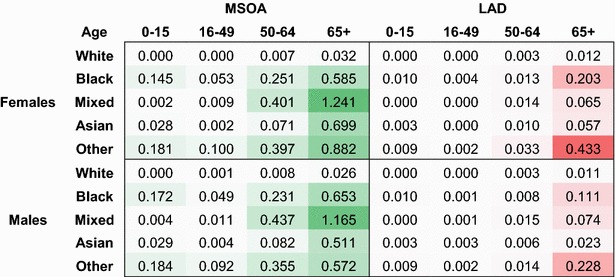
Between-area variance in odds of LLTI by demographic group for LADs and MSOAs *Source:* Authors’ calculations, 2011 census table DC3201EW [39]

Cells are shaded according to the decile corresponding to their value

This pattern differs substantially across minority ethnic groups. In the case of ethnic minorities in general, it seems that between-area differences in prevalence are strong for younger groups; even age groups 0–15 exhibit high dispersion in the case of categories ‘Black’ and ‘Other’. We also find higher between-area variance estimates at the MSOA level for these groups: while for the White group, the between-MSOA variance in odds of LLTI is on average two to three times the between-LAD variance, for most other cross-classifications the variance is multiplied by a factor of five to ten.

For both LADs and MSOAs, highest levels of autocorrelation are measured using the three-nearest matrix $\cdot$**.B3** (see Table 4). Similar measurements taken for higher values of *k* (up to 10 neighbours), not reported in the table, confirmed that increasing the number of neighbours only reduces Moran’s *I* estimates. Estimates for White populations show that odds for older age groups exhibit higher levels of spatial autocorrelation than younger groups. In other words, the spatial clustering of poor health is higher for older age groups. Around retirement age a final wave of intra-national migrations emphasises the clustering of people by health.

**Table 3:**
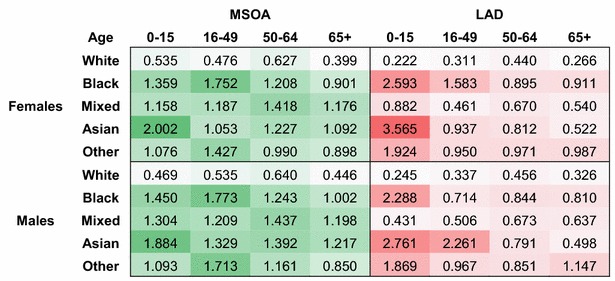
Between-area coefficients of variation for odds of LLTI by demographic group for LADs and MSOAs *Source:* Authors’ calculations, 2011 census table DC3201EW, [39, 60]

Cells are shaded according to the decile corresponding to their value

Interestingly, there is no evidence of the same pattern occurring for Other ethnic groups. On the contrary, the older the individuals reporting an LLTI, the less they are found to cluster in areas. This implies that odds of poor health for ethnic minorities are not only more dispersed than those of White people; they are also less predictable or, in spatial terms, more random. None of the matrices tested in this investigation uncovered substantial spatial structure in the patterns of illness experienced by ethnic minorities, and these structures are very different from those of White populations. We hypothesise that such heterogeneity relates to the presence of stronger socio-economic differences across space for ethnic minorities. In these circumstances, it is unlikely that borrowing strength from the structure exhibited by White populations would help make precise inferences about the health of other populations. There is more potential in using other information such as ethnic density data to reduce the variability in the model, as we show in the next section.

These descriptive estimates also provide indications regarding best fitting adjacency matrices. In the case of LADs, levels of autocorrelation measured using the ‘migration neighbourhoods’ **L.C**$\cdot$ and **L.D**$\cdot$ are lower than with more traditional matrices. Table 4 only reports results for row-standardised, ranked neighbours matrix **L.D3**, because the specification of **L.C***k* (binary weights) did not perform as well. In addition, sensitivity analyses found that the age categories included to generate those migration neighbourhood matrices did not have a strong influence on measures of spatial autocorrelation. More research on age-specific adjacency matrices could refine this observation.

**Table 4:**
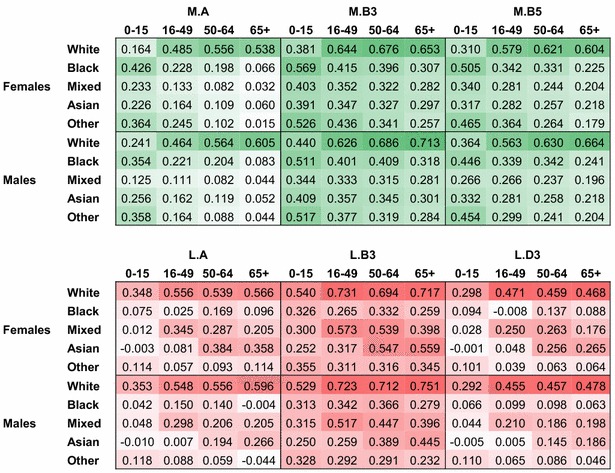
Moran’s *I* statistics of spatial autocorrelation in odds of LLTI by adjacency matrix and demographic group *Source:* Authors’ calculations, 2011 census table DC3201EW [39], Office for National Statistics migration and digital boundary data [60, 62]

Cells are shaded according to the decile corresponding to their value

We conclude from this exploratory work that levels of dispersion in odds of LLTIs, although comparable between sexes, are very dissimilar depending on age and ethnic groups. They may require separate treatment when it comes to their modelling and prediction. Descriptive estimates of autocorrelation provide a strong suggestion that the three-nearest neighbours method $\cdot$**.B**$\cdot$ is likely to be the most efficient since it captures highest levels of homogeneity in odds of LLTI. This finding is consistent across all demographic cross-classifications.

### Modelling with covariates: area classifications and data on ethnicity

We now examine the residual geographical variance in odds of LLTI once contextual information (area classification, ethnic density, mortality rates, and health service data) is introduced in a multivariate framework. We seek to establish whether this contextual information predicts the spatial structure in residuals $\varvec{\upsilon }$, that is to say, shrinks their variance $\sigma ^2$. In this section, we build a series of models predicting LLTI prevalence for LADs exclusively, since they are the level at which contextual data is most commonly available. We begin by introducing some disaggregation using the 2001 National Statistics area classification of English local authorities produced by cluster analysis [63]. This allows us to treat LADs differently according to the following typology;

- Cities and Services; London Suburbs; London Cosmopolitan (reference category)
- London Centre
- Prospering UK
- Coastal and Countryside
- Mining and Manufacturing.

Welsh contextual data being unavailable for LADs, a coarser specification involving a single dummy variable reflecting higher odds of poor health in Wales is retained. To reflect hypotheses of an ethnic density effect in the literature [64], the census estimate of the proportion of the district population identifying as the same ethnicity is incorporated as a covariate for those ethnic groups where such an addition improves the model fit. This is the case for all groups but Black and White populations. With other groups, this covariate improves fit as measured by the AIC and substantially reduces the between-area variance (−12 % for Asian and Other, −7 % for Mixed). This forms specifications for a baseline model (*M0*) fitted separately on data for each of the five ethnic groups (see Table 5). Area-level residuals exhibit mild autocorrelation (Moran’s *I* comprised between 0.3 and 0.6).

**Table 5:** Regression coefficients: baseline models

M0	White			Black			Asian			Mixed			Other		
	*b*	SE	*p*	*b*	SE	*p*	*b*	SE	*p*	*b*	SE	*p*	*b*	SE	*p*
(Intercept)	$-$4.046	0.025	<.001	$-$4.179	0.034	<.001	$-$4.493	0.059	<.001	$-$3.911	0.054	<.001	$-$4.239	0.049	<.001
Male	$-$0.080	0.001	<.001	$-$0.054	0.007	<.001	$-$0.248	0.005	<.001	0.138	0.009	<.001	$-$0.089	0.012	<.001
Aged 16–49	0.914	0.003	<.001	0.816	0.012	<.001	0.658	0.009	<.001	0.980	0.012	<.001	0.924	0.023	<.001
Aged 50–64	2.036	0.003	<.001	1.975	0.013	<.001	2.466	0.009	<.001	2.342	0.015	<.001	2.417	0.024	<.001
Aged 65+	3.179	0.003	<.001	3.232	0.013	<.001	3.623	0.009	<.001	3.201	0.016	<.001	3.353	0.025	<.001
Wales	0.274	0.047	<.001	0.191	0.080	0.017	$-$0.028	0.079	0.727	0.269	0.056	<.001	0.181	0.080	0.024
Lond. centre	$-$0.255	0.076	0.001	0.491	0.097	<.001	$-$0.019	0.115	0.872	0.069	0.085	0.416	0.165	0.103	0.108
Prospering	$-$0.341	0.029	<.001	$-$0.219	0.040	<.001	$-$0.337	0.056	<.001	$-$0.351	0.041	<.001	$-$0.296	0.047	<.001
Coastal	$-$0.181	0.040	<.001	0.147	0.069	0.033	$-$0.316	0.079	<.001	$-$0.075	0.059	0.206	$-$0.097	0.077	0.208
Mining	0.163	0.037	<.001	0.008	0.054	0.876	0.034	0.069	0.620	0.088	0.053	0.093	0.024	0.061	0.698
% same ethnicity							1.887	0.297	<.001	$-$6.016	1.329	<.001	6.477	1.634	<.001
$\sigma ^2$	0.041	0.202		0.062	0.248		0.092	0.303		0.042	0.205		0.051	0.227	
Shapiro–Wilks	0.996		0.519	0.994		0.148	0.995		0.362	0.997		0.724	0.989		0.009
Moran’s *I*	0.511		<.001	0.431		<.001	0.550		<.001	0.503		<.001	0.394		<.001
AIC	97,530			15,517			21,760			16,112			12,298		

### Local mortality and hospitalisation data

We continue with contextual information on mortality and hospitalisations which are expected to be associated with some of the unobserved risk factors modelled through random effects until now. We aim to test whether this information absorbs either between-area heterogeneity or its spatial structure.

Existing evidence [14, 19, 65] demonstrates that individuals are very likely to report an LLTI if they have had or are about to seek a medical diagnosis. In addition, there is a well-known association at the population level between self-reported poor health and local mortality rates [22]. Though non-linear, this association has been exploited for small area estimation using bivariate life table models [20] and relational logistic models [66]. The bivariate response model, relevant for the data at hand, gave a particularly poor fit and was immediately discarded. Instead age-standardised mortality rates (SMRs) from death registrations [67] were transformed through *Z*-standardisation and used as a straightforward covariate. Models (*M1*) (see Table 6) result from best model selection among a range of specifications for each ethnic group separately. We compared sex-specific SMRs, overall SMRs, and interaction with gender dummies. In the case of Black populations, no association with mortality was found. Gains in terms of reduction of between-area variance in random intercepts are important, especially in the case of Mixed ethnic groups, where $\sigma ^2$ is almost halved.

**Table 6:** Regression coefficients: testing models with LAD-level mortality SMRs as predictors of LAD-level prevalence of LLTI

M1	White			Black			Asian			Mixed			Other		
	*b*	SE	*p*	*b*	SE	*p*	*b*	SE	*p*	*b*	SE	*p*	*b*	SE	*p*
(Intercept)	$-$4.046	0.024	<.001	$-$4.189	0.035	<.001	$-$4.305	0.037	<.001	$-$4.210	0.024	<.001	$-$4.167	0.039	<.001
Male	$-$0.095	0.001	<.001	$-$0.054	0.007	<.001	$-$0.248	0.005	<.001	0.137	0.009	<.001	$-$0.090	0.012	<.001
Aged 16–49	0.914	0.003	<.001	0.814	0.012	<.001	0.658	0.009	<.001	0.979	0.012	<.001	0.934	0.023	<.001
Aged 50–64	2.036	0.003	<.001	1.974	0.013	<.001	2.466	0.009	<.001	2.344	0.015	<.001	2.431	0.024	<.001
Aged 65+	3.179	0.003	<.001	3.231	0.013	<.001	3.624	0.009	<.001	3.204	0.016	<.001	3.362	0.025	<.001
Wales	0.270	0.046	<.001	0.184	0.080	0.022	$-$0.088	0.075	0.243	0.248	0.046	<.001	0.132	0.082	0.106
Lond. centre	$-$0.253	0.074	0.001	0.500	0.097	<.001	0.090	0.109	0.412	0.063	0.064	0.325	0.392	0.094	<.001
Prospering	$-$0.330	0.028	<.001	$-$0.195	0.045	<.001	$-$0.348	0.048	<.001	$-$0.052	0.029	0.071	$-$0.290	0.046	<.001
Coastal	$-$0.173	0.039	<.001	0.153	0.069	0.026	$-$0.477	0.066	<.001	0.169	0.040	<.001	$-$0.168	0.073	0.021
Mining	0.162	0.036	<.001	$-$0.012	0.055	0.822	$-$0.295	0.056	<.001	0.137	0.034	<.001	$-$0.125	0.058	0.032
SMR$^\ddag$				0.028	0.021	0.180	0.191	0.021	<.001	0.180	0.013	<.001	0.091	0.022	<.001
Male SMR$^\ddag$ $\times$ male	0.033	0.002	<.001												
Female SMR$^\ddag$ $\times$ female	$-$0.014	0.002	<.001												
$\sigma ^2$	0.039	0.198		0.061	0.247		0.081	0.285		0.024	0.155		0.056	0.237	
Moran’s *I*	0.512		<.001	0.419		<.001	0.456		<.001	0.439		<.001	0.354		<.001
AIC	95,594			15,517			21,726			15,981			12,225		
Reduction in AIC (M0)	1936			0			34			131			73		

*SMR* directly standardised mortality rate

$$^\ddag$$ Variable *Z*-standardised

While mortality data does help predict local prevalence of LLTI, it arguably remains distantly related to chronic morbidity amongst the living. We compare its predictive power with that of indirectly standardised ratio of emergency admissions (SARs) for 2008–2013 [68] on the one hand, and elective admissions [69] on the other hand. This is with the hypothesis that prevalence of LLTI and rates of hospitalisation share common determinants (socio-economic characteristics, lifelong exposure to health determinants). For all ethnicities, rates of emergency admissions are found to be associated with larger regression coefficients and improvements in fit. They are thus selected as the preferred covariate. We then test interaction effects between (a) sex and age variables, (b) mortality and (c) emergency admissions proceeding by backward elimination based on the best sets of covariates, leading us to the set of final models (*M2*) reported in Table 7.

**Table 7:** Regression coefficients: final models predicting LAD-level prevalence of LLTI

M2	White			Black			Asian			Mixed			Other		
	*b*	SE	*p*	*b*	SE	*p*	*b*	SE	*p*	*b*	SE	*p*	*b*	SE	*p*
Intercept	$-$4.121	0.019	<.001	$-$4.187	0.035	<.001	$-$4.528	0.053	<.001	$-$4.086	0.045	<.001	$-$4.398	0.050	<.001
Male	$-$0.095	0.001	<.001	$-$0.054	0.007	<.001	$-$0.233	0.006	<.001	0.137	0.009	<.001	$-$0.089	0.012	<.001
Aged 16–49	0.888	0.003	<.001	0.814	0.012	<.001	0.589	0.011	<.001	0.981	0.012	<.001	0.954	0.023	<.001
Aged 50–64	1.968	0.003	<.001	1.947	0.014	<.001	2.353	0.011	<.001	2.304	0.016	<.001	2.466	0.025	<.001
Aged 65+	3.150	0.003	<.001	3.181	0.014	<.001	3.554	0.010	<.001	3.183	0.016	<.001	3.381	0.025	<.001
Wales	0.344	0.034	<.001	0.222	0.081	0.007	$-$0.050	0.072	0.491	0.275	0.047	<.001	0.178	0.079	0.024
Lond. Centre	$-$0.104	0.056	0.062	0.526	0.096	<.001	0.100	0.101	0.321	0.140	0.063	0.028	0.180	0.096	0.060
Prospering	$-$0.135	0.024	<.001	$-$0.173	0.046	<.001	$-$0.114	0.054	0.034	$-$0.106	0.036	0.003	$-$0.091	0.052	0.078
Coastal	$-$0.018	0.030	0.549	0.184	0.070	0.008	$-$0.201	0.072	0.005	0.103	0.049	0.035	$-$0.002	0.075	0.977
Mining	0.092	0.027	<.001	$-$0.012	0.054	0.819	$-$0.066	0.061	0.278	0.043	0.041	0.292	$-$0.008	0.058	0.886
% same ethnicity							1.898	0.260	0.000	$-$3.493	1.029	0.001	8.926	1.598	<.001
SAR$^\dag$	0.004	0.001	<.001	0.003	0.002	0.068	0.002	0.002	0.302	0.003	0.001	0.001	0.005	0.002	0.005
SAR$^\dag$ $\times$ male	0.001	0.000	<.001				$-$0.003	0.001	<.001						
SAR$^\dag$ $\times$ 16–49	0.004	0.000	<.001				0.003	0.001	<.001						
SAR$^\dag$ $\times$ 50–64	0.010	0.000	<.001				0.002	0.001	0.015				$-$0.004	0.001	<.001
SAR$^\dag$ $\times$ 65+	0.004	0.000	<.001	0.002	0.001	0.040									
SMR$^\ddag$				$-$0.062	0.029	0.030	0.061	0.028	0.032	0.098	0.018	<.001	0.147	0.030	<.001
SMR$^\ddag$ $\times$ male							0.037	0.008	<.001						
SMR$^\ddag$ $\times$ 16–49							0.028	0.012	0.020	0.119	0.011	<.001	$-$0.152	0.012	<.001
SMR$^\ddag$ $\times$ 50 $-$ 64				0.092	0.010	<.001	0.178	0.012	<.001	0.086	0.013	<.001			
SMR$^\ddag$ $\times$ 65+				0.121	0.013	<.001	0.101	0.010	<.001						
Male SMR$^\dag$ $\times$ male	0.027	0.002	<.001												
Female SMR$^\dag$ $\times$ female	$-$0.010	0.002	<.001												
$\sigma ^2$	0.021	0.146		0.059	0.243		0.068	0.260		0.022	0.147		0.042	0.205	
Shapiro–Wilks	0.994		0.192	0.996		0.423	0.995		0.314	0.997		0.863	0.987		0.003
Moran’s *I*	0.555		<.001	0.412		<.001	0.449		<.001	0.410		<.001	0.344		<.001
AIC	85,779			15,239			20,925			15,837			12,040		

*SAR* indirectly standardised emergency admission ratio, *SMR* directly standardised mortality rate

$^\dag$ Variable centred around 1.00

$$^\ddag$$ Variable *Z*-standardised

Overall, for White populations, the new specifications reduce the AIC by over 9800, and the between-area residual variance by 45 %. The effect is less marked for ethnic minorities. The between-area residual variance remains stable for Black populations and is cut by about 20 % for other minorities. Emergency hospitalisations exhibit a strong association with morbidity rates and make the biggest improvement to the models. English areas with observed emergency admissions in excess of 10 % relative to the expected number of admissions (based on age-specific rates of admission for England overall) exhibit odds of LLTI for White persons aged 50–64 years on average 16 % higher compared to areas in line with England’s overall admissions rate. Models remain very different across ethnic groups and the association with admissions rates is weaker for non-White population. Disaggregation of admissions statistics by ethnic group could yield stronger associations in future investigations.

Residuals of each of the final models were examined in detail. Table 8 shows that residuals correlate only very weakly across ethnic categories. This constitutes further evidence that the spatial structure is specific to each of those population groups. Autocorrelation statistics confirm that accounting for differences in mortality and hospitalisation rates does not reduce the spatial autocorrelation in residuals. It reduces the random variability across LADs substantially without offsetting the extent to which deviations of a district’s odds of LLTI from the mean correlate with the deviation measured in neighbouring LADs. From the viewpoint of predictive modelling, it constitutes an advantage; introducing area-level covariates does not reduce the potential to borrow information from neighbouring areas using relevant autoregressive model specifications. In addition, area-level predictors did not lead to important outliers emerging which could signify local departures from the global association with mortality and hospitalisation rates. Aside for individuals from Other ethnic minorities, there is strong evidence both from normal Q–Q plots and Shapiro–Wilks tests that residuals follow a normal distribution.

**Table 8:**
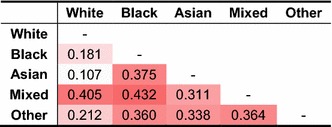
Matrix of pairwise correlation in random intercepts between models (*M2*)

Cells are shaded according to the decile corresponding to their value

**Fig. 1:**
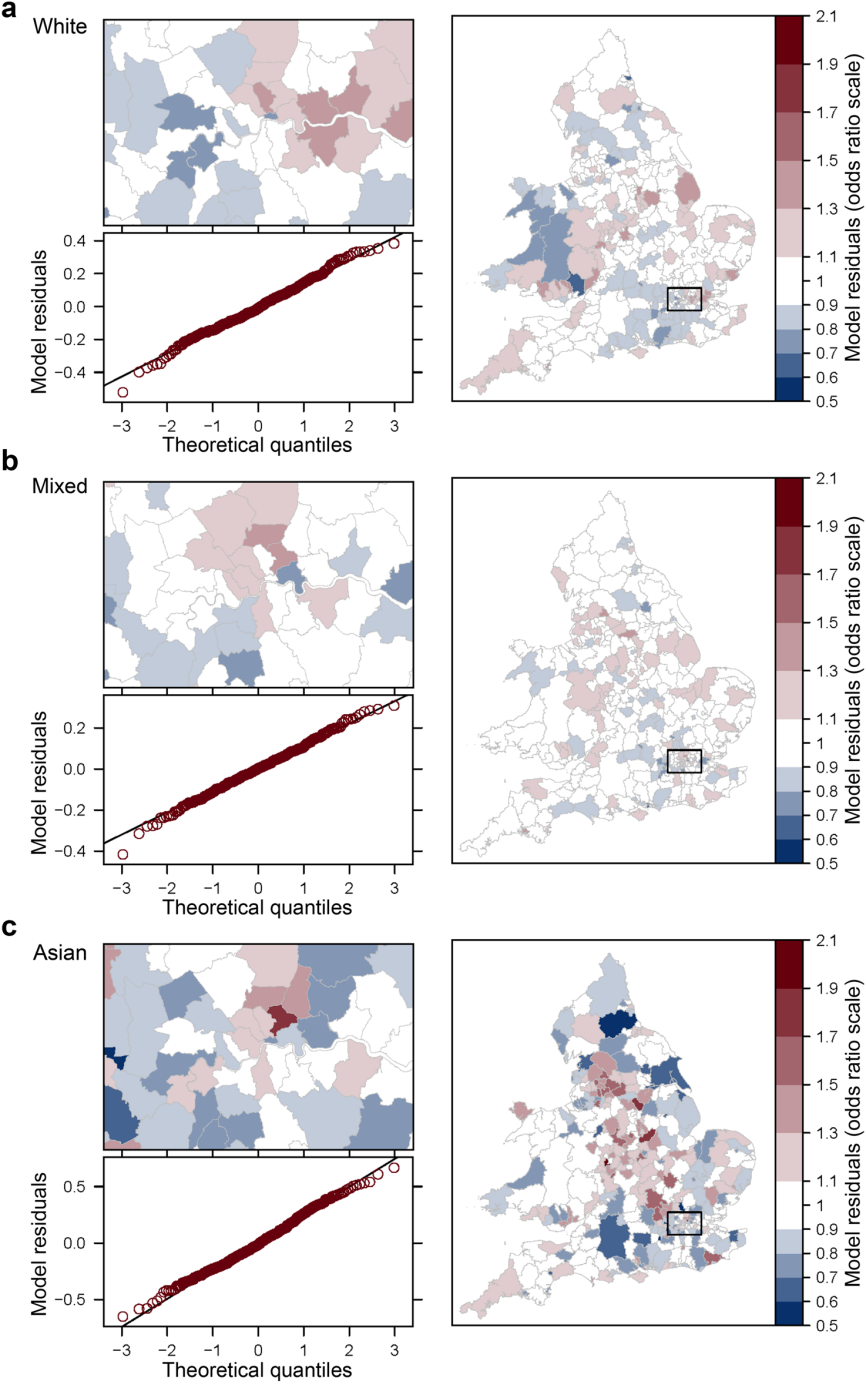
Model (*M2*): Q—Q plots of area residuals against a normal distribution and maps of transformed residuals $e^{\upsilon _d}$ (odds ratio scale) for White (**a**), Mixed (**b**) and Asian (**c**) populations. *Plots* Residuals of model (*M2*) are compared to a theoretical normal distribution with the same mean and standard deviation to assess normality. *Choropleths* Model residuals are converted on the odds ratio scale using the exponential function to map heterogeneity in odds of LLTI across areas once differences in covariates are taken into account. Shades of *red* (*blue*) signal areas where the prevalence of LLTI is *higher* (*lower*) than expected given their population age, area classification and local rates of emergency hospitalisations

**Fig. 2:**
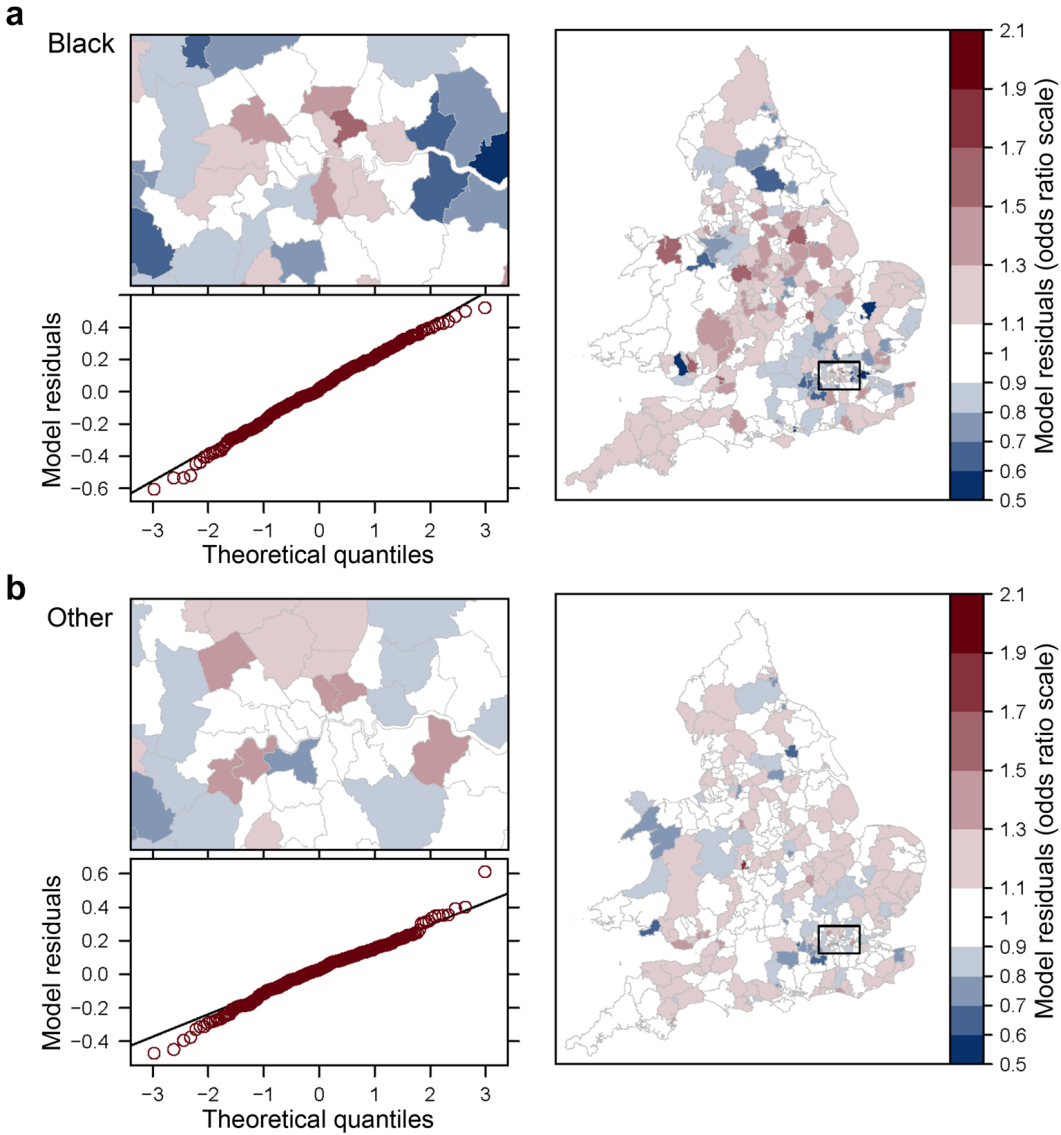
Model (*M2*): Q—Q plots of area residuals against a normal distribution and maps of transformed residuals $e^{\upsilon _d}$ (odds ratio scale) for Black (**a**) and Other (**b**) populations. *Plots* Residuals of model (*M2*) are compared to a theoretical normal distribution with the same mean and standard deviation to assess normality. *Choropleths* Model residuals are converted on the odds ratio scale using the exponential function to map heterogeneity in odds of LLTI across areas once differences in covariates are taken into account. Shades of *red* (*blue*) signal areas where the prevalence of LLTI is *higher* (*lower*) than expected given their population age, area classification and local rates of emergency hospitalisations

**Fig. 3:**
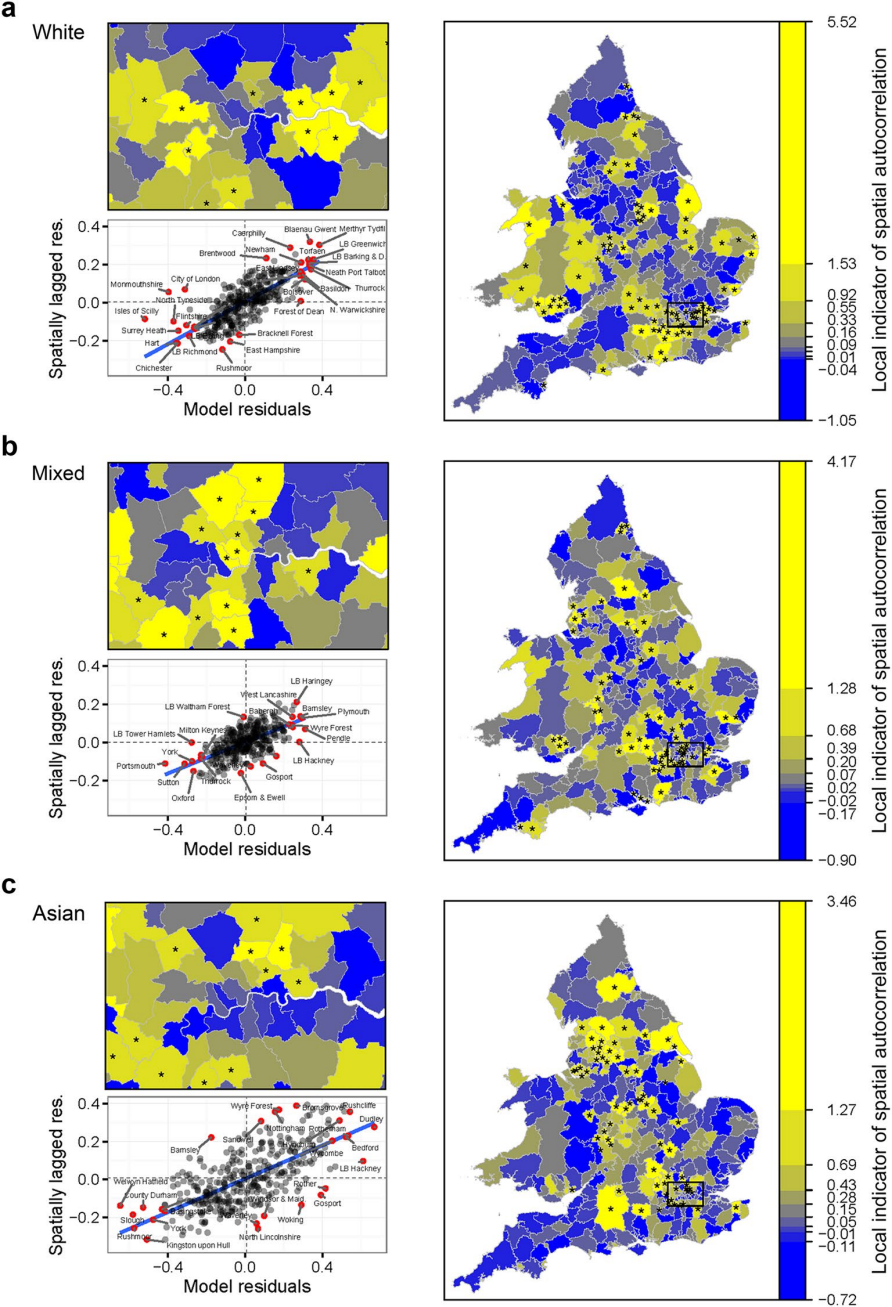
Model (*M2*): Maps of LISA with significant clusters (*asterisks*) and *Moran scatterplots* of area residuals for White (**a**), Mixed (**b**) and Asian (**c**) populations. *Moran scatterplots* Global spatial clustering of LLTI is represented graphically as the relationship between area residuals (on the logit scale) and the spatially lagged area residuals. Some neighbourhoods exhibit higher-than-average clustering and appear above the line of best fit. Significant clusters are marked with a *red dot*. *Choropleths* Shades of *yellow* indicate areas with a *high* LISA, while shades of *blue* indicate areas with a *low* LISA. Statistically significantly higher-than-average LISAs are marked with an *asterisk* (*) and indicate presence of a statistically significant spatial cluster at the 95 % confidence level

**Fig. 4:**
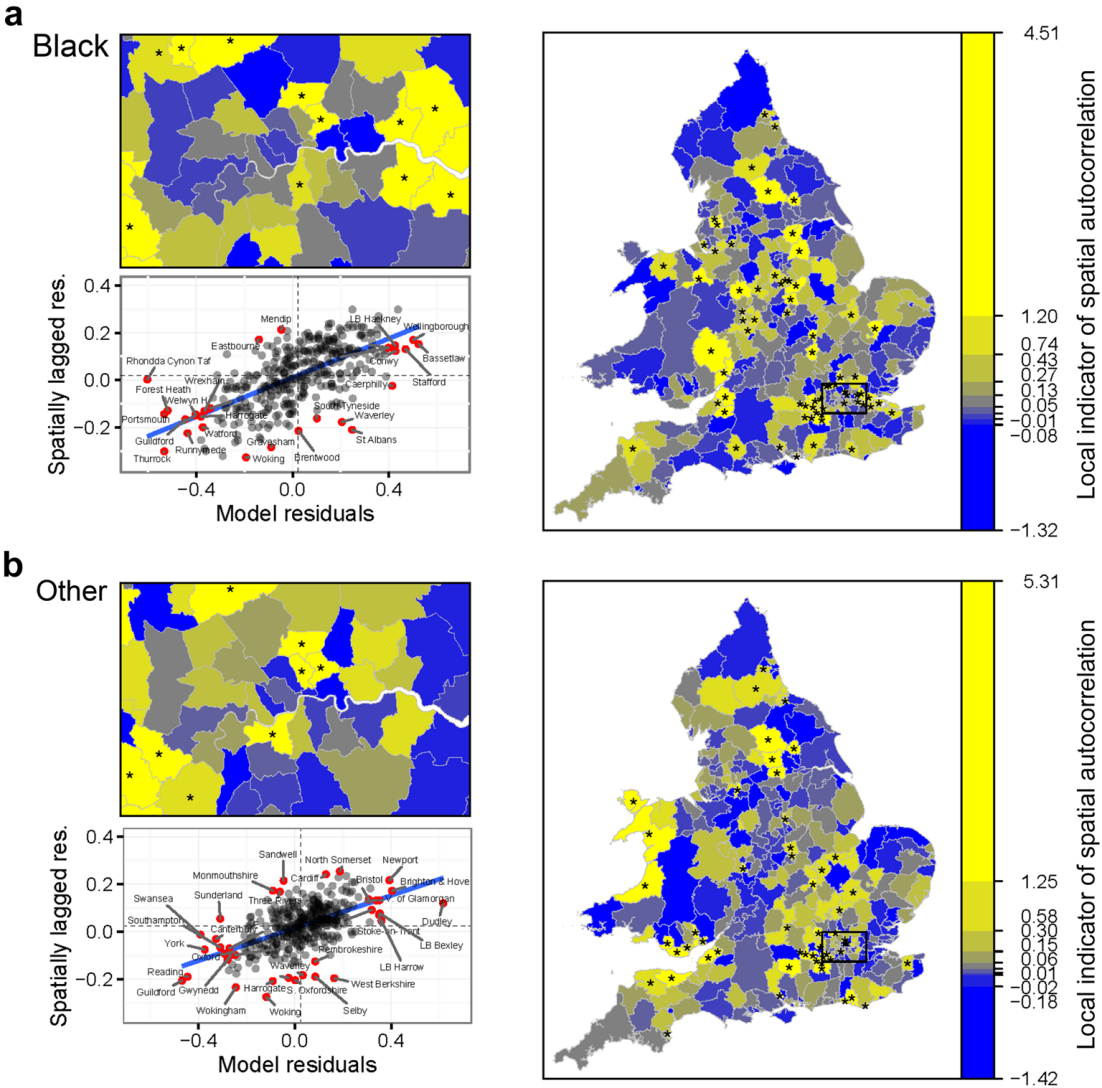
Model (*M2*): Maps of LISA with significant clusters (*asterisks*) and *Moran scatterplots* of area residuals for Black (**a**) and Other (**b**) populations. *Moran scatterplots* Global spatial clustering of LLTI is represented graphically as the relationship between area residuals (on the logit scale) and the spatially lagged area residuals. Some neighbourhoods exhibit higher-than-average clustering and appear above the line of best fit. Significant clusters are marked with a *red dot*. *Choropleths* Shades of *yellow* indicate areas with a *high* LISA, while shades of *blue* indicate areas with a *low* LISA. Statistically significantly higher-than-average LISAs are marked with an *asterisk* (*) and indicate presence of a statistically significant spatial cluster at the 95 % confidence level

Figures 1 and 2 present a series of maps of final model residuals (on the odds ratio scale), which illustrate by how much the fixed part of the model should be multiplied in order to reach the census estimate of odds of LLTI. Unshaded areas indicate predictions falling within $+/-$10 % of the census estimate. Red shades signal LADs where the fixed part of the model underestimates odds by more than 10 % while blue shades LADs where it overestimates odds by over 10 %. With Moran’s *I* estimates close to 0.5, we conclude that half of the deviation between odds for a given district and the national mean is on average shared by its three nearest neighbours. Figures 3 and 4 examine the local contribution of each clique of LADs towards the global measure of spatial autocorrelation. LISAs are calculated, regressed against the model residuals and plotted for each ethnic group separately. Each of the bottom left quadrants signals statistically significant outliers in red, which can be regarded as area residuals which exhibit significant higher or lower similarity with their three nearest neighbours than average, and therefore have particular leverage of the global level of autocorrelation. Together with the maps, it becomes apparent that the chosen modelling and spatial specifications leave important clusters of unexplained risk factors, which are dissimilar across ethnic groups. The Asian model in particular exhibits a lot of heterogeneity in the strength of spatial dependence between LADs, with very strong clusters emerging for instance in parts of Lancashire, Merseyside and Yorkshire, Nottingham and Leicester, as well as North East and South West London boroughs.

## Discussion

Previous work on the 2011 census has highlighted the presence of a strong spatial structure in univariate morbidity statistics [70]. Analysis reported in this paper presents a deeper examination of multivariate aspects of this spatial dependence. Descriptive estimates suggest that the variability in odds of poor health across groups and places is larger than can be expected from just looking at crude prevalence estimates. For instance, area effects are often thought to correlate strongly across age groups, as reflected in random walk priors proposed by Congdon [25]. Our analysis looking at ethnicity provides strong evidence that patterns of spatial dependency in the odds of LLTI differ substantially across ethnic groups. The covariate-adjusted spatial structure of LLTI in White people only moderately correlates with that for Mixed ethnic groups. Structures of LLTI of all other groups correlate very weakly with each other. Descriptive estimates for ethnic minorities also reveal that levels of spatial autocorrelation are higher for young people, in constrast with the increased autocorrelation measured among older age groups in White people. Reasons for this difference are unclear and call for further research. One can hypothesise that cohort exposure is different for ethnic minorities and that older people reporting a minority ethnic identity have more diverse histories of exposure to risk factors, or are not affected by the same health-selecting processes of residential segregation.

The rationale for stratifying our analysis by ethnic group resides in the substantial interest in understanding variations in health care need across different population groups. Since the LLTI indicator has been used as a proxy for health care need [71], it is interesting to understand whether care needs of different ethnic groups are stable across different places. Our findings are in line with Finney’s work [72] and confirm that knowledge on patterns and determinants of local ethnic health gaps remains insufficient. Overall, disaggregation of ethnicities reveals more variation than would arise purely out of the combination of local age characteristics and chance. Our finding is also consistent with previous investigations by Shouls et al. [21, 24] relying on factor analysis to classify LADs with respect to known area-level aggregate health estimates. Our analysis of spatial autocorrelation patterns confirms that even when accounting for other common population health measurements such as rates of hospitalisation and mortality, which we assume capture important unobserved risk factors, the significant remaining between-area heterogeneity still exhibits strong, almost unaffected spatial patterns in a way that is specific to each ethnic group. This is a sign of very different health needs and has been identified as an important area of current research [73].

We can draw implications for predictive modelling. In addition to measuring disparities in health needs which are not already contained in mortality and hospitalisation statistics, the distinct spatial pattern of overdispersion in the final model confirms the importance of reviewing assumptions on random effects in multilevel health models. While the assumption of spatially independent residuals may be sufficient in many descriptive epidemiology studies, it introduces risks of substantial variation and clustering in the quality of small area prediction across space, especially in the presence of underpowered sample data. This has seldom been raised as a validity issue with disease prevalence prediction models [74] though the importance of testing for the existence of significant between-area heterogeneity was noted by Datta et al. [75].

This paper gives a practical illustration of the implications of assuming independence of random effects across areas. In our results, the degree to which the fixed part of the model underestimates or overestimates odds of LLTI is highly dependent on error in neighbouring areas. It implies that, in the absence of sufficient individual-level auxiliary data (e.g. from a census) or area-level predictors (e.g. statistics on health utilisation, social or occupational characteristics), there is a greater need to explicitly model these spatial structures not just using covariate adjustments, but also incorporating spatial information explicitly into regression models. The literature has identified several routes for doing so [76]. A common stochastic approach is the use of spatial or conditional autocorrelation functions, by introducing spatial matrices into the model’s covariance structure [77]. A competing approach is the use of spatial trend surfaces (polynomial functions of the geographic coordinates) [35], or Euclidean distance matrix eigenvalues [78–80] as regressors in a standard generalised linear model.

All these techniques presuppose that the structure underpinning spatial processes in the data is well understood. In addition to reviewing existing structures defined around both adjacency and proximity (*k*-nearest method), the purpose of this paper was to test the relevance of an alternative definition of spatial structure based on residential migration. Levels of autocorrelation reached with this method indicate that while it is not the best fitting method for LLTI, it does capture a non-negligible spatial interaction.

Incorporating spatial information explicitly into regression models requires good prior knowledge regarding the study outcome. The main benefit of using a large population source, such as the census in this paper, is to be able to conduct additional tests on local levels of autocorrelation. In our case, substantial local clusters were apparent in the residuals for the Asian model, suggesting that the range of covariates used was particularly inappropriate to predict local odds, even once global autocorrelation was taken into account. This constitutes further evidence of the need to better understand the spatial structure of chronic conditions.

Our study indicates that small area estimation remains a data intensive task. It remains difficult to predict LLTI with simple models without introducing socio-economic information on local populations from a source such as the census [81]. Looking at between-area heterogeneity (Figs. 1, 2), it is apparent that geographical inequalities remain which prove difficult to predict. With these models, about 46.8 % of LADs require an adjustment of the fixed part of the model by at least $+/-$10 % for White populations while 15.8 % require an adjustment of at least $+/-$20 % in order to reach the actual odds of LLTI. The latter figure is of 9.5 % for Mixed ethnic groups, 15.8 % for Other ethnic groups, 33.6 % for Black populations and as high as 40.2 % for Asian populations. A reasonably large sample of data is required for every area of interest in order to reach a precise predictor of random parameters $\varvec{\upsilon }$. This has implications for power calculations to obtain good quality empirical best predictors in presence of such residual variability. Moreover there are also questions around the properties of synthetic estimators, which only make use of the fixed part of the prevalence model, in situations where the between area residual variance $\sigma ^2$ is not negligible. Such estimators currently underpin the majority of UK disease prevalence models. These issues point to the importance of tests for heterogeneity recently examined by Datta et al. [75] and Molina et al. [82].

While models can help produce estimates for small populations, hypothesis testing can prove limiting. The range of possible small area model specifications is virtually limitless. Shortcomings are likely to arise especially in cases where models demonstrate similar levels of unexplained variance and spatial clustering in their random part. This highlights the importance of large-scales studies such as censuses in providing reliable auxiliary information for small groups.

## Conclusions

The key contributions of this paper relate to (1) new descriptions of the spatial structure in LLTI both in terms of dispersion and autocorrelation and (2) implications for predictive modelling and small area estimation.

With regard to the first point, we present greater disaggregation than previous investigations in this area [21, 24, 70] and emphasise the importance of ethnicity and alternative conceptualisations of ‘spatial structure’. We provide a systematic analysis of best-fitting spatial structures and give an applied example of a new method to build adjacency matrices using migration data. Further research could examine the predictive power of disaggregating migration interaction according to demographic characteristics (age and ethnicity being strong determinants in spatial terms). It would also be worth considering spatial interaction beyond the notion of symmetry, by examining hypotheses where *A* being a ‘neighbour’ of *B* does not imply the reciprocal. Alternative approaches have proposed treating spatial weights as random parameters to be estimated rather than as fixed data [83]. This may reduce subjectivity in model specification, arguably at a certain computational and precision cost.

Our second contribution concerns the applied relevance of the paper to concerns related to predictive modelling, particularly around planning efficient small area estimation strategies. In the UK, there is sustained interest in information for small geographical areas [84], in a context where local population health surveys have almost entirely disappeared due to rising fieldwork costs and falling nonresponse [85]. Persistent, and often widening health inequalities are a concern internationally [86–88], with improvements in small area public health monitoring among major policy recommendations to tackle such problems [89].

Subnational monitoring of morbidity levels raises particular statistical challenges. Our results show that geographical variability in the odds of LLTI are greater than expected not only from sampling error and differences in local populations’ age distributions, but also in relation to levels of mortality and healthcare utilisation. Odds of LLTI also exhibit a larger between-area variance for ethnic minorities compared to White populations.

From a methodological viewpoint, we acknowledge limitations commonly encountered in disease mapping. In addition to data limitations themselves (insufficient disaggregation of age bands, quality issues usually expected from hospital data), this research relies on complex models. Only taking into account main fixed effects of model (*M2*), the number of candidate models is $2^{12}$. When taking into account the different types of hospital and mortality covariates, the number of possibilities rises to millions. Specifications with complex random effects and spatial autocorrelation structures could in addition be considered, raising this number even higher. Overall, this concern, well-identified in predictive modelling [90–92], represents a challenge in transparency and reproducibility of public health information.

Overall, our results emphasise the importance of detailed contextual information on population characteristics and spatial structures in the production of working models that can be trusted to hold for the whole population. Modelling techniques can be applied which make use of the spatial clustering illustrated in this paper to improve prediction. Yet, like empirical prediction, these require access to good-quality survey data with individual geographical identifiers (for instance postcode sectors) for all of the targeted small areas. In the future, geographic masking [93] may offer safer alternatives in situations where geographical identifiers are too disclosive to be released. This study also highlights the importance of local health care statistics to improve the predictive capability of models. Further disaggregation of these data sources by ethnic group and groups of medical conditions at the local level is likely to help improve future disease prevalence models.

## Additional files

10.1186/s12942-016-0057-5 R syntax. This syntax contains commands to download Office for National Statistics internal migrations datasets, process them and compute an interaction matrix of size 348 $\times$ 348 following methods $\cdot$.C*k* and $\cdot$.D*k* (see Table 1). The output can be used as a spatial weights matrix in spatial analyses.
10.1186/s12942-016-0057-5 Sensitivity analyses. This document reports on the effect of different minimum age thresholds on resulting migration interaction matrices, taking the example of neighbours allocated to the City of Manchester, the City of Nottingham and the London Borough of Barnet under different thresholds.

## Abbreviations

AIC: Akaike Information Criterion
LAD: local authority district
LISA: local indicator of spatial autocorrelation
LLTI: limiting long-term illness
MSOA: middle layer super output area
Q–Q plot: quantile–quantile plot
SMR: directly age-standardised mortality rate
SAR: indirectly standardised emergency admission ratio
