# Supplementary material for: The spatial structure of chronic morbidity: evidence from UK census returns
Source: Int J Health Geogr. 2016 Aug 24;15(1):30. doi: 10.1186/s12942-016-0057-5 (PMC4997767; doi:10.1186/s12942-016-0057-5)
Supplement: Supplementary file 2 — 10.1186/s12942-016-0057-5 Sensitivity analyses. This document reports on the effect of different minimum age thresholds on resulting migration interaction matrices, taking the example of neighbours allocated to the City of Manchester, the City of Nottingham and the London Borough of Barnet under different thresholds. [file 12942_2016_57_MOESM2_ESM.docx]

## Supplementary material

## Sensitivity testing of age cut-offs for building neighbourhood matrices using migration data

This section reports on sensitivity analysis performed in order to ensure that the intensity of sub-national migration flows for younger residents does not have an excessive influence on the way local authorities are matched with one another to form a neighbourhood matrix based on the number of residents exchanged. Figure 1 below illustrates the spike of migration for individuals aged 16 to 34 years.

1. Intensity of annual intra-national migrations by age in England and Wales 2009-2011 (Source: ONS 2013*b*).

In order to examine how the migration peak influences the ranking of pairwise migration intensity between districts, we generated these ranking separately for residents aged 30+, then 25+, 20+ and finally 15+. Results are reproduced in Table 1 below for the City of Manchester, the City of Nottingham and the London Borough of Barnet. After manual reviews of those matrices we concluded that changing the minimum age only has a moderate effect on the ranking of neighbours.

1. Sensitivity analyses for neighbours allocated to two metropolitan councils and one city council LADs under different age scenarios

|  |  | **Manchester** | | | |  | **Barnet** | | | |
| --- | --- | --- | --- | --- | --- | --- | --- | --- | --- | --- |
| **Min. age** |  | 30 | 25 | 20 | 15 |  | 30 | 25 | 20 | 15 |
| **1^st^ neighbour** |  | Salford | Salford | Salford | Trafford |  | Camden | Camden | Camden | Camden |
| **2^nd^ neighbour** |  | Trafford | Trafford | Trafford | Salford |  | Haringey | Haringey | Haringey | Haringey |
| **3^rd^ neighbour** |  | Stockport | Stockport | Stockport | Stockport |  | Enfield | Brent | Brent | Brent |
| **4^th^ neighbour** |  | Oldham | Oldham | Bury | Bury |  | Brent | Enfield | Enfield | Enfield |
| **5^th^ neighbour** |  | Bury | Bury | Tameside | Tameside |  | Hertsmere | Hertsmere | Harrow | Hertsmere |

|  |  | **Nottingham** | | | |
| --- | --- | --- | --- | --- | --- |
| **Min. age** |  | 30 | 25 | 20 | 15 |
| **1^st^ neighbour** |  | Rugby | Rugby | Erewash | Erewash |
| **2^nd^ neighbour** |  | Erewash | Erewash | Rugby | Rugby |
| **3^rd^ neighbour** |  | N. Warwickshire | N. Warwickshire | N. Warwickshire | N. Warwickshire |
| **4^th^ neighbour** |  | Malvern Hills | Lancaster | Lancaster | Lancaster |
| **5^th^ neighbour** |  | Lancaster | Malvern Hills | Derby | Craven |
